# Supplementary material for: Silica Nanospheres Coated Silver Islands as an Effective Opto-Plasmonic SERS Active Platform for Rapid and Sensitive Detection of Prostate Cancer Biomarkers
Source: Molecules. 2022 Nov 13;27(22):7821. doi: 10.3390/molecules27227821 (PMC9697738; doi:10.3390/molecules27227821)
Supplement: Supplementary file 1 [file molecules-27-07821-s001.zip › molecules-1957169-supplementary.pdf]

Supplementary Materials

Silica Nanospheres Coated Silver Islands as an Effective opto-plasmonic SERS Active Platform for Rapid and Sensitive Detection of Prostate Cancer Biomarkers

Anamika Pandey[†], Subhankar Sarkar[#], Sumit Kumar Pandey[†] and Anchal Srivastava[†*]

[†]Department of Physics, Institute of Science, Banaras Hindu University, Varanasi-221005, India

[#]School of Computational and Integrative Sciences, Jawaharlal Nehru University, New Delhi-110067, India

*Corresponding author

Email: anchalbhu@gmail.com, anchal@bhu.ac.in

Ph.: +91-9453203122

Synthesis of SiO_2 nanospheres

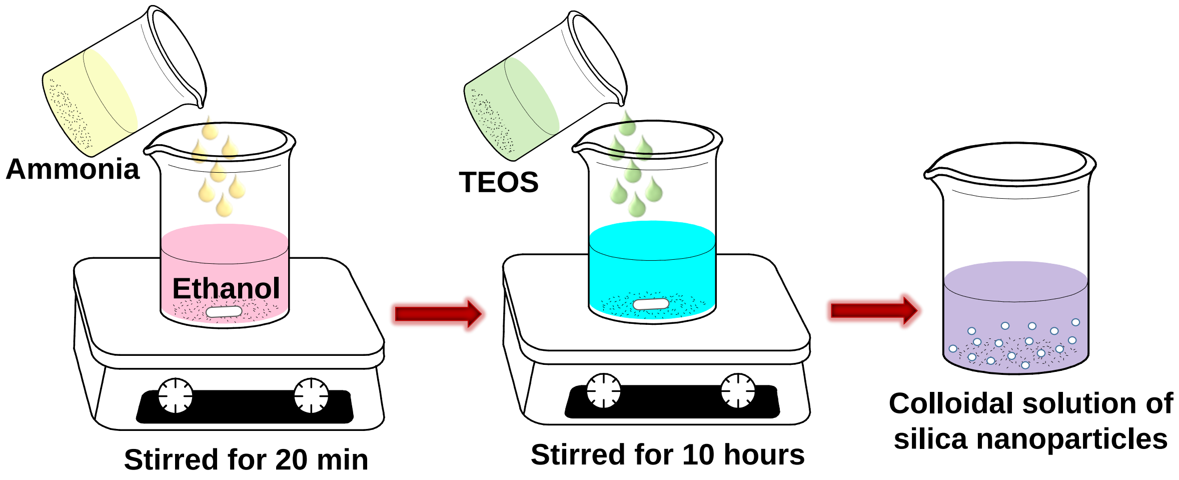

Figure S1: Schematic procedure for the synthesis of silica nanospheres *via* Stober's method.

Fabrication of SERS substrate

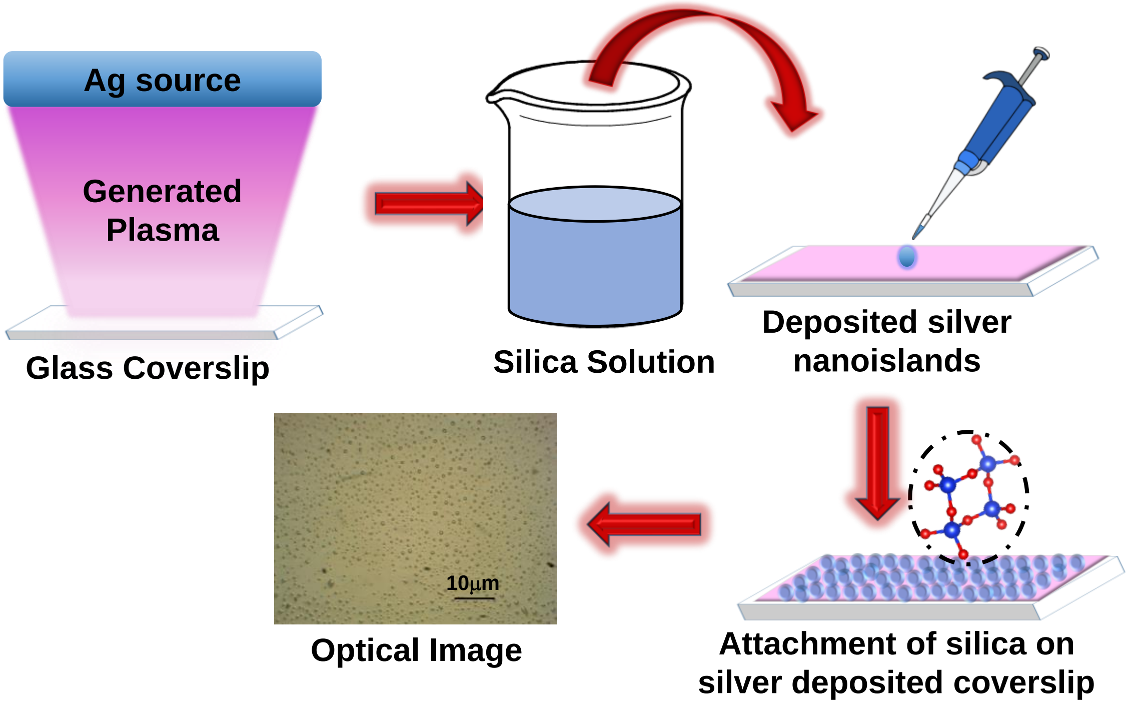

Figure S2: Schematic illustration of the fabrication of SERS substrate with the aid of RF sputtering.

SERS measurements

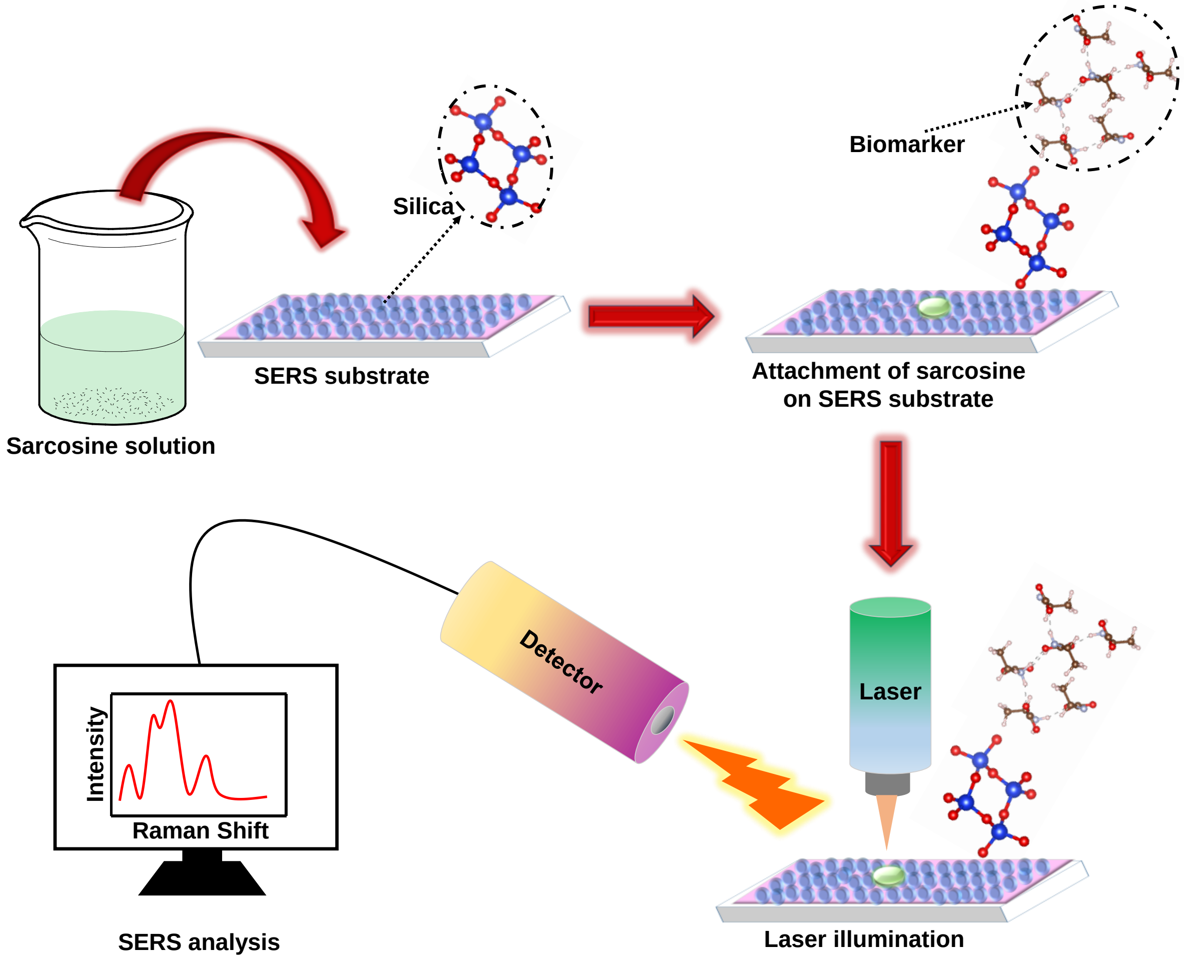

Figure S3: Schematic illustration of SERS analysis of sarcosine.

UV-vis absorption spectra of silica nanospheres and band gap calculation

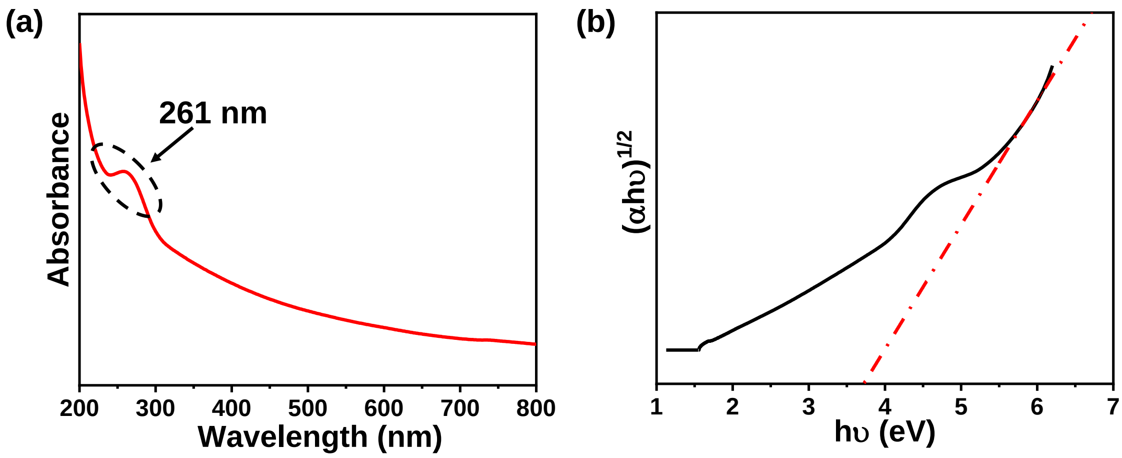

Figure S4: (a) UV-vis absorption spectra of as-synthesized SiO_2 nanospheres. **(b)** the band gap estimation of synthesized SiO_2 nanoparticles using Tauc's plot.

AFM 3D topography of silver nano-islands and silica nanospheres decorated silver nano-islands

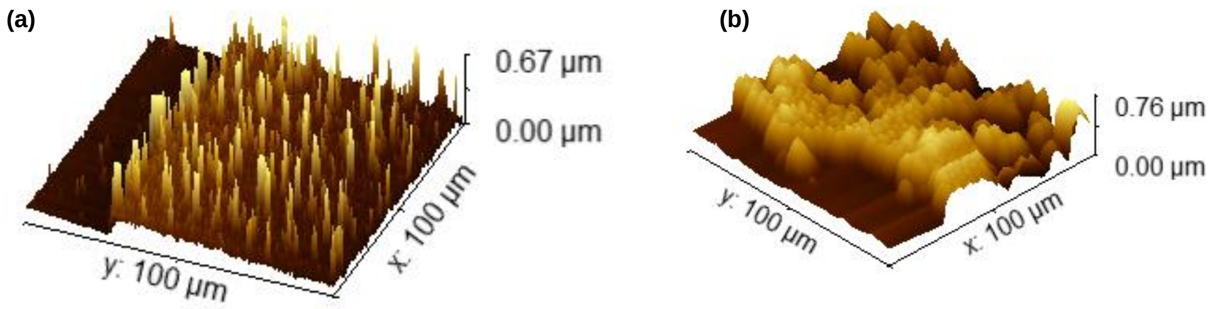

Figure S5: (a) 3D topography of silver nano-islands. **(b)** silica nanospheres decorated silver nano-islands.

Raman spectra of pure bulk sarcosine powder

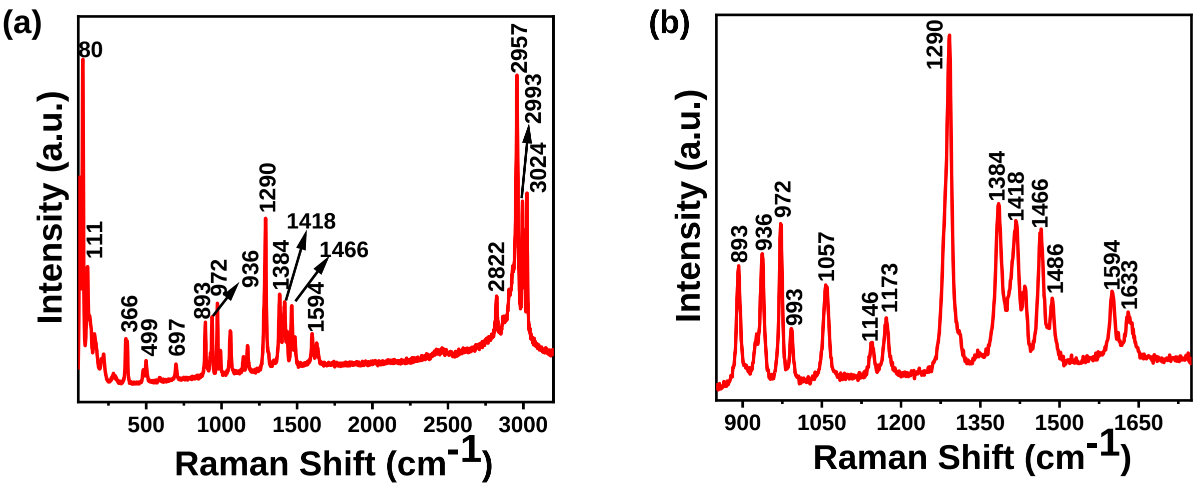

Figure S6: (a) Raman spectra of pure bulk sarcosine powder. **(b)** Raman spectra of pure bulk sarcosine powder within the range of 850 cm^{-1} to 1750 cm^{-1}

Table S1: The chemical structure and various vibrational Raman bands of the prostate cancer biomarker *viz* sarcosine.

	**Peak Position (cm^{-1})**	**Assignment**	**Relative Intensities (a.u.)**
	3024	CH_3 stretching (asymmetric)	8186
	2993	CH_2 stretching (symmetric)	7919
	2957	CH_2 stretching (symmetric)	12767
	2822	CH_3 stretching (symmetric)	4191
	1633	NH_2 deformation, C=O stretching	2315
	1594	COO^- stretching (asymmetric), NH_2 deformation	2674

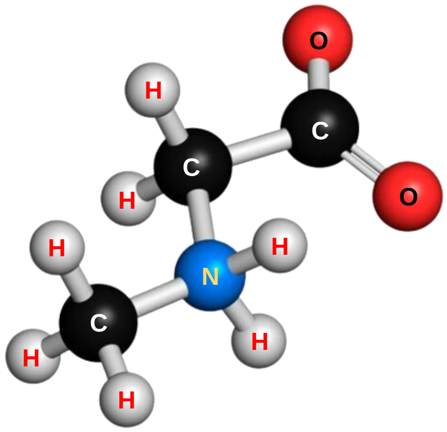	1486	CH_2 rocking and deformation	2579
	1466	CH_3 asymmetric in-plane bending, CH_2 deformation	3771
	1418	COO^- stretching (symmetric), CH_2 deformation	3919
	1384	CH_3 symmetric in-plane bending, CH_2 deformation	4197
	1290	CC stretching	7166
	1173	NH_2 rocking	2206
	1057	CO stretching	2803
	972	CH_2 out-of-plane bending	3860
	936	COO^- deformation, and CH_2 rocking	3336
	893	CO stretching, CC stretching	3119
	697	COO^- deformation	1536
	499	COO^- rocking, C-N deformation	1671
	366	CH_2 torsion	2495

Optical images of the SERS substrate before and after the Raman experiment

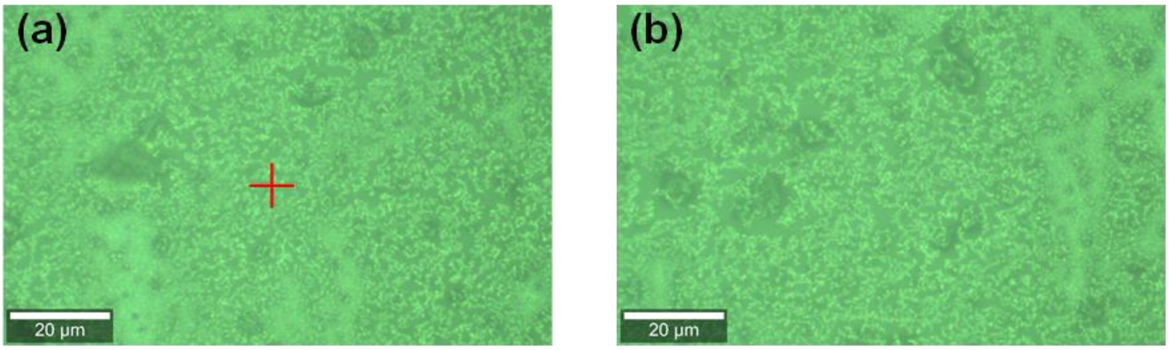

Figure S7: Optical images of SERS substrate **(a)** before Raman experiment **(b)** after Raman experiment.

Raman spectrum of prepared and used SiO_2@Ag SERS substrate

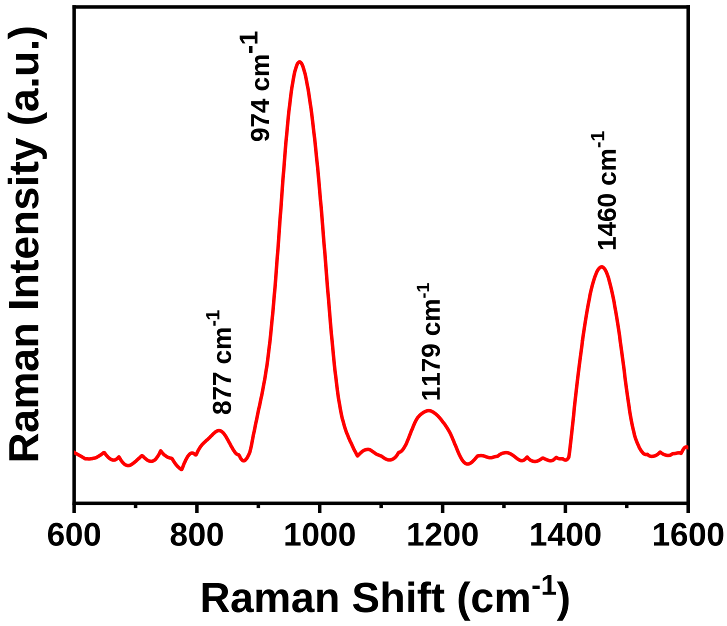

Figure S8: Raman spectrum of prepared and used SiO_2@Ag SERS substrate. The Raman spectrum shows peaks at 877 cm^{-1} and 974 cm^{-1} which corresponds to SiO_2 nanospheres. The peaks at 1179 cm^{-1} and 1460 cm^{-1} correspond to Ag nano-islands.

Calculation of limit of detection (LOD)

LOD of as fabricated SERS substrate has been calculated using the equation (1) given below

$$\text{LOD} = 3.3 \times (\text{SD of intercept/slope}) \tag{1}$$

SD (standard deviation) of intercept can be calculated by using the formula given below in equation (2):

$$\text{SD of intercept} = \text{SE (standard error) of intercept} \times \sqrt{N} \tag{2}$$

here, the value of SE of intercept is 9.847 which has been obtained using the calibration curve. N is no. of the sample, i.e., 5. So, SD can be calculated as:

$$\text{SD of intercept} = 9.847 \times \sqrt{5}$$

$$= 22.017$$

From the calibration curve, the value of slope = 41.182

By substituting the values of SD of intercept and slope in equation (1), LOD can be calculated as:

$$\text{LOD} = 3.3 \times (22.017/41.182)$$

$$= 3.3 \times 0.534$$

$$= 1.76 \text{ nM}$$

Hence, the LOD is found to be 1.76 nM.
